# Integration of audiovisual spatial signals is not consistent with maximum likelihood estimation

**DOI:** 10.1016/j.cortex.2019.03.026

**Published:** 2019-10

**Authors:** David Meijer, Sebastijan Veselič, Carmelo Calafiore, Uta Noppeney

**Affiliations:** Computational Cognitive Neuroimaging Laboratory, Computational Neuroscience and Cognitive Robotics Centre, University of Birmingham, Birmingham, UK

**Keywords:** Multisensory perception, Optimality, Maximum likelihood estimation, Multisensory integration, Spatial ventriloquism

## Abstract

Multisensory perception is regarded as one of the most prominent examples where human behaviour conforms to the computational principles of maximum likelihood estimation (MLE). In particular, observers are thought to integrate auditory and visual spatial cues weighted in proportion to their relative sensory reliabilities into the most reliable and unbiased percept consistent with MLE. Yet, evidence to date has been inconsistent. The current pre-registered, large-scale (N = 36) replication study investigated the extent to which human behaviour for audiovisual localization is in line with maximum likelihood estimation. The acquired psychophysics data show that while observers were able to reduce their multisensory variance relative to the unisensory variances in accordance with MLE, they weighed the visual signals significantly stronger than predicted by MLE. Simulations show that this dissociation can be explained by a greater sensitivity of standard estimation procedures to detect deviations from MLE predictions for sensory weights than for audiovisual variances. Our results therefore suggest that observers did not integrate audiovisual spatial signals weighted exactly in proportion to their relative reliabilities for localization. These small deviations from the predictions of maximum likelihood estimation may be explained by observers' uncertainty about the world's causal structure as accounted for by Bayesian causal inference.

## 1 Introduction

Sensory organs provide the brain with information about the outside world. Information from different senses can be complementary (e.g., an object's shape viewed from the front but haptically explored from the back) or redundant (e.g., the object's location). For example, both visual and auditory modalities provide uncertain information about the spatial position of a mosquito flying in a dimly lit room. In order to obtain the most reliable and unbiased estimate (i.e., an estimate that is associated with the least variance or uncertainty) an observer should integrate redundant sensory information weighted in proportion to their relative reliabilities according to maximum likelihood estimation (MLE) (Ernst & Banks, 2002). Reliability weighted integration according to MLE (i.e., the ‘ideal observer’ model; here simply called ‘MLE model’) thus sets a benchmark of statistically optimal performance against which human behaviour can be compared (Ernst & Bülthoff, 2004).

In their seminal study, Alais and Burr (2004) showed that human audiovisual localization conforms to the predictions of the MLE model. In a 2-interval forced choice (2IFC) localization task, participants were presented a so-called standard stimulus in the middle in one interval and a so-called probe stimulus at various locations along the azimuth in the other interval. Standard and probe were either both auditory or visual or audiovisual. Participants indicated which of the two stimuli (standard or probe) was located more on the left. The reliability of the visual stimuli, a low contrast Gaussian blob, was manipulated by blurring (i.e., increasing its size), whereas the reliability of the auditory stimuli, short click sounds, was kept constant. By introducing a small, unnoticeable spatial conflict between the auditory and visual components of some of the audiovisual stimuli Alais and Burr were able to determine the relative weights that participants assigned to the auditory and visual signals during audiovisual integration. As predicted by the MLE model, observers integrated auditory and visual signals in proportion to their relative reliabilities that were computed from the unisensory auditory and visual conditions (n.b. the reciprocal of response variance corresponds to the perceived reliability). They assigned a weight to the visual signal that increased with the visual reliability. Moreover, the variance (i.e., unreliability) of the audiovisual spatial estimates was smaller than the variances of the unisensory auditory and visual spatial estimates. Again, the audiovisual variance was closely predicted by the MLE model based on the variance of unisensory percepts.

However, the conclusions of Alais and Burr (2004) are not supported in a related study of audiovisual spatial integration by Battaglia, Jacobs, and Aslin (2003). In this study, participants' integrated sensory signals weighted by their reliability, yet the visual weights were significantly higher than predicted by the MLE model. In our own lab, we have recently observed similar visual overweighting during audiovisual spatial integration (here described as our pilot data, see Appendix A). Battaglia et al. (2003) have argued that visual overweighting may result from human observers imposing a prior on the sensory reliabilities based on their everyday experiences: i.e., in most situations the visual spatial signal is far more reliable than the auditory spatial signal. Such priors are not incorporated in the MLE model. Alais and Burr (2004) briefly mention in the discussion that their participants were trained extensively on the auditory localization task, which may potentially have taught participants to trust their auditory sense more, leading to a stronger auditory weight. Yet, a life-long prior on the sensory modalities is just one of many possible accounts of why human behaviour diverges from MLE predictions (for a recent review, see Rahnev & Denison, 2018). Most importantly, in the multisensory and wider perception literature the findings by Alais and Burr are interpreted and generally cited as evidence that human observers integrate sensory signals or cues in line with the MLE predictions. Multisensory integration according to MLE predictions is considered a generic and fundamental mechanism of how human observers integrate information from multiple sources. Therefore, it is important to ascertain that naïve human observers indeed integrate sensory signals from vision and audition weighted in proportion to their relative sensory reliabilities as predicted by the MLE model.

In line with previous research the current study investigated whether human behaviour is consistent with predictions of the MLE model in two steps: First, we investigated whether participants integrated the auditory and visual signals in proportion to their unisensory reliabilities (i.e., we compared empirical and predicted sensory weights). Second, we investigated whether the variance reduction of the audiovisual percept is equal to the MLE predicted variance reduction. Since we found the empirical sensory weights to be significantly different from the MLE-predicted weights we conclude that audiovisual spatial integration for untrained participants is not adequately described by the MLE model.

## 2 Method

### 2.1 Maximum likelihood estimation model

The MLE model makes two key quantitative predictions for observers' integrated audiovisual location estimates. First, an observer should integrate the unisensory location estimates ${\hat{S}}_{A}$ and ${\hat{S}}_{V}$ weighted in proportion to their relative sensory reliabilities:

(1) $${\hat{S}}_{AV}=w_{A}{\hat{S}}_{A}+w_{V}{\hat{S}}_{V}\;\mathrm{with}\;w_{A}=\frac{r_{A}}{r_{A}+r_{V}}=\frac{\frac{1}{{\sigma_{A}}^{2}}}{\frac{1}{{\sigma_{A}}^{2}}+\frac{1}{{\sigma_{V}}^{2}}}\;\mathrm{and}\;w_{V}=\frac{r_{V}}{r_{V}+r_{A}}=\frac{\frac{1}{\sigma_{V}^{2}}}{\frac{1}{{\sigma_{V}}^{2}}+\frac{1}{{\sigma_{A}}^{2}}}$$

where $w_{V}$ and $w_{A}$ are the sensory weights and reliability (r) is the inverse of the sensory variance ($\sigma^{2}$).

Second, the sensory variance of the integrated estimate is predicted to be lower than the sensory variance of either of the unisensory estimates:

(2) $${\sigma_{AV}}^{2}=\frac{{\sigma_{A}}^{2}{\sigma_{V}}^{2}}{{\sigma_{A}}^{2}+{\sigma_{V}}^{2}}<\text{min}\left({\sigma_{A}}^{2},{\sigma_{V}}^{2}\right)$$

This second equation is generally considered the more stringent test for the MLE model, as it confirms that the two unisensory signals are truly integrated on a trial-by-trial basis (i.e., the forced fusion assumption); whereas the first equation may also hold (on average) if ${\hat{S}}_{AV}$ is fully determined by either ${\hat{S}}_{A}$ or ${\hat{S}}_{V}$, but when the choice for either is made probabilistically in proportion to the sensory weights (i.e., ‘cue switching’; Ernst & Bülthoff, 2004).

### 2.2 Experiment overview

This study aimed to examine whether the MLE model accurately predicts the results of untrained participants in an audiovisual localization task that was designed to be nearly identical to the study by Alais and Burr (2004). The most striking difference is that we use only one visual reliability level (but see Section 2.6.2.2), which is individually adjusted for each participant to match his/her auditory reliability level (see Section 2.6.1.3). Matching of the unisensory reliabilities is important in order to maximize the MLE-predicted variance reduction for audiovisual stimuli relative to the most reliable unisensory stimuli (Eq. (2), Section 2.1). This experimental choice was made to optimize the chances of arbitrating between MLE-based integration and ‘cue switching’.

### 2.3 Sample characteristics

The primary outcome measures were two group-level one-sided paired t-tests that assessed the two key MLE predictions (Eqs. (1), (2), Section 2.1) by testing for differences between the empirically determined and MLE-predicted sensory weights and audiovisual variances (see Section 2.9). The null hypothesis stated that the MLE model describes participants' audiovisual integration adequately (i.e., in line with the findings of Alais and Burr (2004) there is no significant difference between MLE predicted and empirical weights or AV variances). Any significant (*p* < .05) difference between predicted and empirical weights/variances indicated that the data were not consistent with the MLE model; as previously reported by Battaglia et al. (2003). For Battaglia et al.’s average effect size (Cohen's d) of .58 (estimated across different stimulus reliability levels; their Fig. 7) an a-priori power analysis revealed that 36 participants were required to obtain high statistical power (1-β = .96, α = .05, $d_{z}$ ≥ .58; as computed with G*Power 3.1; Faul, Erdfelder, Buchner, & Lang, 2009; www.gpower.hhu.de). Based on this power analysis, we decided to include thirty-six participants in the final analysis and results (i.e., excluded participants were replaced until 36 complete data sets were obtained, see Section 2.11).

All participants were university students with reportedly normal hearing, (corrected to) normal vision and no history of neurological or psychiatric disorder. Participants provided informed consent and were compensated by means of study credits or cash.^2^ The study was approved by the human research review committee of the University of Birmingham (approval numbers ERN_11-0470AP4 & ERN_15-1458P^3^).

### 2.4 Stimuli

The visual stimulus was a greyscale circular blob with a bivariate Gaussian amplitude envelope. Its size (defined by the 2D Gaussian's standard deviation, $\sigma_{blob}$; symmetrical in all directions) was adjusted individually for each observer to equate the unisensory spatial uncertainties for visual and auditory spatial estimates (Section 2.6.1.3). Visual stimuli were presented for a duration of 16.7sec (msec) in low-contrast (20 cd/m^2^ in its centre) on a darker grey background (15 cd/m^2^).^4^

The auditory stimulus was a 16.7 msec burst of white noise (70 dB SPL),^5^ which included a 5 msec on/off ramp. To create virtual spatial sound sources along the azimuth, the auditory signal was convolved with standardised head-related transfer functions (Gardner & Martin, 1995; http://sound.media.mit.edu/resources/KEMAR.html).

### 2.5 Two interval forced choice paradigm

**Fig. 1:**
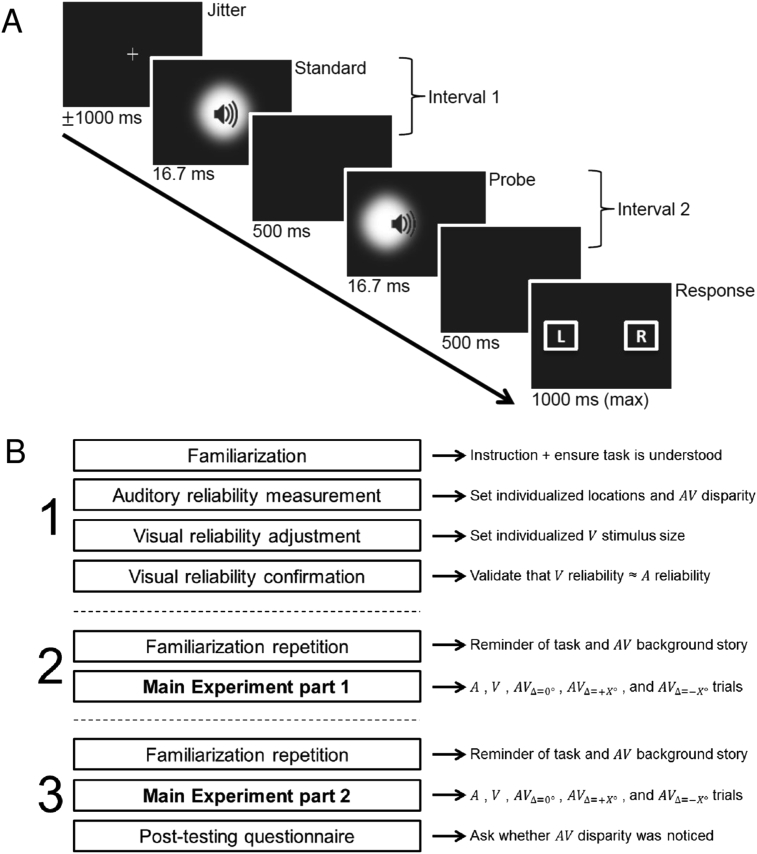
Trial structure for the audiovisual localization task (Panel A) and full experimental procedure (Panel B). A. A jittered pre-stimulus time period, in which participants fixated a cross in the middle, was followed by two intervals, each of which consisted of a stimulus and a subsequent blank period. The stimuli in the two intervals were either both auditory or visual or audiovisual (the latter is shown here). The first stimulus, the ‘standard’, was always presented in the middle. The second stimulus, the ‘probe’, was presented at one of thirteen locations along the azimuth. An audiovisual probe could be spatially congruent, or incongruent (with a small spatial conflict between the auditory and visual signals; as shown here). After the second interval, two rectangles appeared on the screen to prompt participants to indicate via a two choice key press whether the location of the probe was left or right of the standard. B. The experiment included three sessions on three separate days (vertically numbered in the figure). In session 1 we individually (for each participant) adjusted the probe locations and AV spatial disparity (Section 2.6.1.2) and the spatial perceptual reliability of the visual signal to match the spatial perceptual reliability of the auditory signal. The visual reliability was adjusted by changing the size of the visual stimulus (i.e., $\sigma_{\mathrm{blob}}$; see Section 2.6.1.3). At the end of session 1, we validated that auditory and visual reliabilities were approximately equal (Section 2.6.1.4). In sessions 2 and 3, the probe locations, AV spatial disparity and visual stimulus size were set to the levels defined in session 1 and they were not further adjusted during the main experiment (but see Section 2.6.2.2). All tasks, throughout all three sessions made use of the trial structure as described in Panel A.

All tasks presented auditory, visual or audiovisual stimuli in a two-interval-forced choice (2IFC) paradigm. Fig. 1A provides a trial overview.

Participants were presented on each trial with a standard in the first interval and a probe in the second interval to avoid sequential order effects that may have affected the estimation of the slope parameters (Dyjas, Bausenhart, & Ulrich, 2012). The interstimulus interval was 500 msec. Probe and standard within a trial were of the same sensory modality, i.e., both auditory (A), visual (V) or audiovisual (AV). The standard was always presented at 0° visual angle along the azimuth, whereas the probe was presented at a location that is selected with equal probability from thirteen possible locations that were determined individually for each participant based on his/her auditory just noticeable difference (JND; see Section 2.6.1.2), unless mentioned otherwise (see Sections 2.6.1.1, 2.6.1.2, 2.6.1.3). Critically, while the AV standard was always spatially congruent, the AV probe was either spatially congruent (i.e., $AV_{\Delta =0{}^{\circ}}$) or spatially incongruent with a small, so-called non-noticeable audiovisual spatial disparity ΔAV (i.e., $AV_{\Delta =+X{}^{\circ}}$ or $AV_{\Delta =-X{}^{\circ}}$, where the visual stimulus' location was moved by $+\frac{1}{2}\Delta AV$ and the auditory stimulus was moved by $-\frac{1}{2}\Delta AV$; n.b. the size of ΔAV was adjusted individually, see Section 2.6.1.2). 500 msec after probe offset two rectangles were presented to prompt participants to report whether the probe was left or right of the standard. Observers indicated their response by pushing a button with their left or right index finger (maximum response time = 1 sec; the prompt disappeared after a response was given). The trial onset asynchrony was jittered between 750 and 1250 msec. Prior to standard onset, participants fixated a central grey cross (1° diameter) with luminance equal to the centre of the visual stimuli.

The sensory modality of the trials was blocked (A, V or AV, in pseudorandom order) and indicated to participants prior to block begin. In AV blocks, the order of the congruent and incongruent trials was randomized (main experiment only, Section 2.6.2.2).

### 2.6 Experimental procedure

The study consisted of three 2.5 h sessions that were performed on three separate days. In the following we will describe the series of experimental parts in the first, second and third sessions; as shown in Fig. 1B.

#### 2.6.1 First session

##### 2.6.1.1 Familiarization

Brief familiarization runs were introduced at the beginning of each session to ensure that participants understood and were familiar with the task. They also mitigated learning effects and reduced variability of perceptual reliability across sessions. Participants were provided with the background story that the AV stimulus was to be considered the result of somebody hitting the back of the screen with a metal stick^6^ (the visual blob representing the stick's imprint during the hit) in order to enhance observer's so-called ‘forced fusion’ assumptions that the AV’s auditory and visual component signals come from a common source (Alais & Burr, 2004). Participants then completed a short familiarization series that included A, V and $AV_{\Delta =0{}^{\circ}}$ trials (in session one: 5 trials x 3 conditions x 12 locations: ±1°, ±4°, ±7°, ±10°, ±13°, ±15°; with highly reliable visual stimuli: $\sigma_{blob}$ was pseudorandomized between 2° and 8°). After every response participants were given immediate corrective feedback, i.e., a green/red circle was presented on the screen to indicate a correct/incorrect response (200 msec duration).

##### 2.6.1.2 Auditory reliability measurement

This experimental part consisted of two parts.^7^ Participants first completed a series of A trials (20 trials x 13 locations: 0°, ±1°, ±2°, ±3°, ±5°, ±7°, ±10°). Participants that obtained an accuracy of less than 90% for those forty trials on which the probe was presented at ±10° azimuth were excluded at this stage (i.e., they did not participate in the main experiment). For each participant we fitted a psychometric function to the fractions of ‘perceived right’ across the thirteen probe locations (see Section 2.8). The auditory spatial uncertainty, expressed as the just noticeable difference (JND), is given by the inverse of the fitted slope parameter $\left(JND=\frac{1}{\beta }\right)$. These individual auditory JNDs were used at three levels in the experiment: (i) probe locations, (ii) visual reliability and (iii) spatial disparity.

i. Probe locations: We set the probe locations for all subsequent parts of the study in a subject-specific fashion according to locations=(0,±0.5,±1,±1.5,±2,±2.5,±3)∗JND (rounded to .5° under the constraint that the 13 locations were unique). This procedure ensures that the psychometric functions of each participant were sampled at comparable probabilities of ‘right’ responses thereby providing more reliable estimates of slope, PSE and lapse rate parameters (Wichmann & Hill, 2001a).
ii. Visual reliability: We adjusted the reliability, i.e., size of the visual Gaussian blob individually for each participant to match the auditory perceptual reliability (see Section 2.6.1.3).
iii. AV spatial disparity: Previous studies have demonstrated that observers' sensitivity to detecting whether or not sensory signals come from a common source and should be integrated according to forced fusion assumptions depends on sensory reliability (Rohe & Noppeney, 2015a). Based on a power analysis simulation (see Appendix B), we set AV disparity equal to one auditory JND individually for each participant (i.e., ΔAV=±JND; conform recommendations by Rohde, van Dam, & Ernst, 2016). The power analysis simulation (Appendix B) suggested that a spatial disparity of one auditory JND allows one to reveal with high statistical power (1–β = .95) that the empirical weight deviates from the MLE-predicted weight by approximately .06 or more. Yet, this limited spatial disparity also maximized the probability that participants integrated sensory signals into one unified audiovisual percept according to forced fusion strategies rather than take into account the causal structure of the sensory signals as accommodated by more complex causal inference models (see Körding et al., 2007, Shams and Beierholm, 2010, Rohe and Noppeney, 2015a, Rohe and Noppeney, 2015b, Rohe and Noppeney, 2016; and further discussions in Appendix B).

The second part of the ‘auditory reliability measurement’ is a refinement of the first part's measurement by using the individualized JND-based locations (see point i above), thereby ensuring an adequately measured auditory JND. Participants completed a second series of A trials (20 trials x 13 individualized locations). The new auditory JND that was obtained from a second fitted psychometric function replaced the JND from the first measurement. This second auditory JND was used in all further tasks (see points i, ii, and iii above).

##### 2.6.1.3 Visual reliability adjustment

Using adaptive staircases we adjusted the size of the Gaussian blobs (defined by $\sigma_{blob}$, Section 2.4) such that the reliability of the V and A spatial perceptual estimates were equated individually for each subject. First, we obtained observer's auditory localization performance for locations at (±0.5,±0.85,±1.2)∗JND from the fitted psychometric function (Section 2.6.1.2). Using two unisensory visual interleaved adaptive staircases for each of these three location pairs we adjusted the size of the Gaussian blob such that the fraction ‘perceived right’ in the visual trials matched the target fractions estimated from the psychometric function of the auditory condition [$\sigma_{blob}$ starting values: 2° and 40°; $\sigma_{blob}$ decreased after each incorrect response and increased after U consecutive correct responses (U = 1, 2, 4 for the three location pairs, respectively) with up/down step sizes $\left(\Delta^{+}/\Delta^{-}\right)$ weighted according to: $fraction\;correct={\left(\frac{\Delta^{-}}{\Delta^{-}+\Delta^{+}}\right)}^{\frac{1}{U}}$; Kingdom & Prins, 2016]. The adaptive staircases were terminated after 30 reversals. For each staircase $\sigma_{blob}$ was computed pooled over the last 20 reversals. For each participant we identified which of the six staircases provided the estimate that was most distant from the pooled $\sigma_{blob}$ across all six staircases. To attenuate effects of potential outliers, we discarded this estimate^8^ and then computed the final pooled $\sigma_{blob}$ across the remaining five staircases (i.e., $\sqrt{\frac{1}{n}*\sum \left({\sigma_{blob}}^{2}\right)}$, with n = 5 staircases * 20 reversals).

##### 2.6.1.4 Visual reliability confirmation

To validate that V and A variances were successfully equated, participants completed a series of 260 V trials (20 trials x 13 individualized locations) with a constant visual stimulus size ($\sigma_{blob}$ as determined in Section 2.6.1.3) and 260 V trials (20 trials x 13 individualized locations) with variable visual stimulus sizes (selected pseudo-randomly between $\frac{1}{2}*\sigma_{blob}$ and $2*\sigma_{blob}$)^9^. The V trials with constant stimulus size were presented interleaved with the variably sized V trials. Importantly, the variably sized V trials were not analysed (i.e., trials of ‘no interest’) and only served to ensure similar conditions as in the main experiment (see Section 2.6.2.2).

For each participant we fitted a psychometric function to the fractions of ‘perceived right’ for the V trials with constant stimulus size, across the thirteen probe locations, and the variance was computed from the fitted psychometric function (see Section 2.8). If (i) the difference between the variances obtained from this V and the previous A (Section 2.6.1.2) psychometric functions was too large [i.e., if it led to a MLE-predicted multisensory variance reduction of less than one third of the smallest unisensory variance: ${\sigma_{AV,mle}}^{2}>\frac{2}{3}*\text{min}\left({\sigma_{A}}^{2},{\sigma_{V}}^{2}\right)$ according to Eq. (2), Section 2.1], or if for either of the two psychometric functions (A or V) (ii) the lapse rate was larger than .06 (Wichmann & Hill, 2001b) or (iii) the goodness-of-fit was insufficient (see Section 2.10), then participants were considered to be unreliable with respect to their localization performance and therefore excluded (and replaced) from the study at this stage.

#### 2.6.2 Second and third session

##### 2.6.2.1 Familiarization repetition

At the beginning of sessions 2 and 3, participants were reminded of the background story (as described in Section 2.6.1.1) and took part in a short familiarization run (with feedback after every trial, see Section 2.6.1.1) to minimize variability in perceptual reliability and task performance across sessions [5 trials x 3 conditions x 12 locations: (±0.5,±1,±1.5,±2,±2.5,±3)∗JND with visual reliability similar to the main experiment (Section 2.6.2.2): $\sigma_{blob}$ was pseudorandomized between $\frac{1}{2}*\sigma_{blob}$ and $2*\sigma_{blob}$].

##### 2.6.2.2 Main experiment

Participants completed 520 trials (40 trials x 13 individualized locations) for each of the 5 main conditions (A, V, $AV_{\Delta =0{}^{\circ}}$, $AV_{\Delta =+X{}^{\circ}}$, $AV_{\Delta =-X{}^{\circ}}$; where X° is the individualized audiovisual disparity ΔAV, see Section 2.6.1.2; i.e., 520 × 5 = 2600 ‘trials of interest’) as well as an additional 520 V trials and 3 × 520 = 1560 $AV_{\Delta =0{}^{\circ}}$ trials (i.e., 2080 trials of ‘no interest’). Critically, in half of the V and AV trials (i.e., ‘trials of interest’) the visual stimulus size $\left(\sigma_{blob}\right)$ was constant and defined based on the results of session 1, such that visual and auditory reliabilities were equated. In the other half of the V and AV trials (i.e., ‘trials of no interest’) the visual stimulus size was variable and selected pseudo-randomly between $\frac{1}{2}*\sigma_{blob}$ and $2*\sigma_{blob}$. These latter ‘trials of no interest’ were not analysed. They were included to ensure that observers could not rely on a stored set of sensory weights, but needed to compute the sensory weights on a trial-by-trial basis. The AV trials of no interest were all spatially congruent.

The main experiment (4680 trials spread over two days) was divided into 20 short A, V and AV blocks. A blocks included 26 trials, V blocks 52 trials and AV blocks 156 trials. The number of trials varied across sensory modalities because the A reliability level was fixed for auditory stimuli. By contrast, for half of the V and AV trials (i.e., trials of interest) the visual reliability was fixed, while it was variable for the other half of the visual trials (i.e., trials of no interest). Further, AV stimuli were presented three times as frequent as V stimuli, because AV stimuli were presented without audiovisual conflict (i.e., spatially congruent; $AV_{\Delta =0{}^{\circ}}$), with a positive audiovisual conflict $\left(AV_{\Delta =+X{}^{\circ}}\right)$ and with a negative audiovisual conflict $\left(AV_{\Delta =-X{}^{\circ}}\right)$. The blocks of the different sensory modalities (A, V, AV) were presented in pseudorandom order and equally split across the second and third session (i.e., main experiment part 1 and part 2, see Fig. 1B). Importantly, only data from this main experiment was used to assess whether participants integrated the AV signals as predicted by MLE. Thus, A, V and AV conditions were controlled for stimulus exposure and experimental duration (n.b. the unisensory localization performances in session 1 or familiarization tasks were not used in the final analysis).

##### 2.6.2.3 Post-testing questionnaire

At the end of the third session participants completed a short questionnaire. Embedded in general questions about participants' subjective performance [e.g., “Did you get tired during the experiment and do you think this affected your accuracy?” and “Rate the difficulty of the task (scale 1–10) for the three different stimuli: auditory only, visual only, and audiovisual”] the following important question was asked: “For audiovisual stimuli, did you ever have the impression that the auditory and visual signals did not come from the same location?” Responses to this question served as subjective reports on whether the audiovisual spatial conflict was indeed non-noticeable (i.e., the forced-fusion assumption).

### 2.7 Experimental setup

Participants were seated behind a table in a dark room with their chin on a chinrest placed at a distance of 75 cm from a grey screen (opaque fine PVC fabric; 127.5 cm width × 170 cm height). The visual stimuli were back-projected onto the screen using a 60 Hz DLP projector (BenQ MW529). The sounds were presented by means of headphones (Sennheiser HD 280 Pro) with a playback frequency of 192 kHz. Auditory and visual stimulus presentation was controlled using Psychtoolbox 3.0.12 (Brainard, 1997, Kleiner et al., 2007; www.psychtoolbox.org) running on MATLAB R2016a (www.mathworks.com) with maximum audiovisual asynchronies < 2 msec (100 stimulus presentations, .03 msec mean, .5 msec standard deviation).

Fixation was monitored using a desktop mount Eyelink 1000 eye tracker (www.sr-research.com) that was calibrated before the start of each block of trials.^10^ Trials on which the participant failed to fixate within a 3° radius during a 1 sec period prior to probe onset, or in which blinks were recorded during either of the stimuli presentations, were excluded from further analysis.

### 2.8 Fitting psychometric functions

For each observer, we computed the fraction of ‘perceived right’ for each of the thirteen probe locations (on the horizontal axis *x*), separately for each condition. These thirteen data points per condition can be described by the psychometric function (ψ), a model with three parameters (α, β, λ):

(3) $$\psi \left(x;a,\beta ,\lambda \right)=\lambda +\left(1-2\lambda \right)F\left(x;\alpha ,\beta \right)\;\mathrm{with}\;F\left(x;\alpha ,\beta \right)=\frac{\beta }{\sqrt{2\pi }}\int_{-\infty }^{x}exp\left(-\frac{\beta^{2}{\left(x-\alpha \right)}^{2}}{2}\right)$$

where F(x;α,β) is the cumulative normal distribution, *α* is the mean of the normal distribution (i.e., point of subjective equality, PSE), the so-called slope parameter β is the reciprocal of the participant's spatial uncertainty (i.e., just noticeable difference, $JND=\frac{1}{\beta }$), and λ is the lapse rate (i.e., the probability of an incorrect response independent of probe location x) (Kingdom & Prins, 2016). N.b. The JND in this 2IFC localization task is related to the sensory variance of the stimuli ($\sigma^{2}$) according to: $JND^{2}=2\sigma^{2}$ (i.e., the sensory noise of standard and probe both contribute to the JND).

A psychometric function is ‘fit’ to observers' fraction of ‘perceived right’ responses by adjusting its parameter values (α, β, λ) such that the likelihood of the data is maximized. For this we used the Nelder-Mead optimization algorithm, as implemented in Palamedes toolbox 1.8.2 (www.palamedestoolbox.org). Likelihood (L) is computed as the following product:

(4) $$L=\prod_{i=1}^{N}{\bar{p}}_{i}^{k_{i}}*{\left(1-{\bar{p}}_{i}\right)}^{\left(n_{i}-k_{i}\right)}$$

where ${\bar{p}}_{i}=\psi \left(x_{i};a,\beta ,\lambda \right)$ is the expected probability of observing a ‘right’ response given probe location $x_{i}$ and parameter values for a, β and λ (Eq. (3)); $k_{i}$ is the empirical number of ‘right’ responses out of $n_{i}$ trials, and N is the total number of probe locations.^11^

For analysis of the main experiment, we simultaneously fitted five psychometric functions to the five different conditions: A, V, $AV_{\Delta =0{}^{\circ}}$, $AV_{\Delta =+X{}^{\circ}}$ and $AV_{\Delta =-X{}^{\circ}}$ (i.e., the product of the five likelihoods is maximized), individually for each observer. To avoid biases in the slope parameters (β) by inaccuracies of the estimated lapse rate parameters (λ) for the individual conditions, we constrained the lapse rate parameters to be equal across all five conditions (Kingdom & Prins, 2016; see specifically their Box 4.6 and Section 9.2.5). In other words, we assumed that observers' miss-responses for non-specific reasons such as blinking, inattention etc. would be comparable across conditions. Furthermore, we assumed one common slope parameter for the three (i.e., one congruent, one positive and one negative spatial conflict) AV conditions (N.b. the MLE model predicts equality of the slopes across the AV conditions; it is therefore standard to compute a single AV variance estimate by averaging the variances of the AV conditions; e.g., see Alais & Burr, 2004). Given those parameter constraints we obtained 5 Gaussian means (i.e., α=PSE), 3 Gaussian variances [i.e., $0.5*{\left(\frac{1}{\beta }\right)}^{2}=\sigma^{2}$], and 1 lapse rate parameter (λ) for each observer.

Critically, the results of these psychophysics experiments rely on participants' maintaining attention and being willing to perform this audiovisual location task in a reliable fashion. However, it is unrealistic to expect from participants that their performance, vigilance and internal criteria remain absolutely constant over the duration of five hours of psychophysical testing (across sessions 2 and 3, see Section 2.6.2.2). To account for the non-stationarity in observers' behaviour and the associated overdispersion we have therefore used the betabinomial model (Fründ et al., 2011, Schütt et al., 2016)^12^. The betabinomial model assumes that the response probability (e.g., expected probability ‘right’, $\bar{p}$ in Eq. (4)) is not fixed throughout the entire experiment but a beta-distributed random variable. The variance of the response probability is determined by the scaling factor η (between 0 and 1). In order to fit the betabinomial model, including η, we have used the following equation for the likelihood (instead of Eq. (4); Schütt et al., 2016):

(5) $$L=\prod_{i=1}^{N}\frac{B\left(k_{i}+\eta^{\prime }{\bar{p}}_{i},\;n_{i}-k_{i}+\eta^{\prime }\left(1-{\bar{p}}_{i}\right)\right)}{B\left(\eta^{\prime }{\bar{p}}_{i},\;\eta^{\prime }\left(1-{\bar{p}}_{i}\right)\right)}\;\mathrm{with}\;\eta^{\prime }=\frac{1}{\eta^{2}}-1$$

where B denotes the beta function and the other parameters are the same as in Eq. (4). To clarify, only one η parameter is fitted per participant (similar to the shared lapse rate parameter λ); i.e., i=1…N denotes a unique combination of probe location and condition (N = 65; 5 conditions × 13 probe locations).

To ensure adequate performance and model fits, we excluded (and replaced) participants if (i) the lapse rate was greater than .06 (Wichmann & Hill, 2001b) or (ii) the goodness-of-fit was insufficient (see Section 2.10).

### 2.9 Sensory weights and AV variances

The normal distributions' variances of the unisensory conditions (A and V) were used to compute the MLE predictions for the auditory weight ($w_{A}$ in Eq. (1), Section 2.1) and for the variance of the AV percept (${\sigma_{AV}}^{2}$ in Eq. (2), Section 2.1). The empirical auditory weight was computed from the audiovisual conditions with a small spatial cue conflict (i.e., $AV_{\Delta =+X{}^{\circ}}$ and $AV_{\Delta =-X{}^{\circ}}$, with X° equal to the auditory JND, see Section 2.6.1.2):

(6) $$w_{A,emp}=\frac{PSE_{\Delta AV=+X{}^{\circ}}-PSE_{\Delta AV=-X{}^{\circ}}}{2\text{*}|\Delta \text{AV}|}+\frac{1}{2}$$

where the PSEs serve as the means of the location estimates $\hat{S}$ (c.f. Eq. (1), Section 2.1).^13^ Please note that in consistency with previous work Eq. (6) makes the additional assumption that $PSE_{\Delta AV=0{}^{\circ}}=PSE_{A}=PSE_{V}$; i.e., that the spatial bias is equal for AV congruent, A and V conditions (Fetsch, Pouget, DeAngelis, & Angelaki, 2011).

The primary outcome measures of this study were the results of statistical comparisons that investigated whether the i. empirical auditory weight and ii. empirical AV variance were significantly different from the MLE predictions (i.e., $w_{A,mle}$ *vs* $w_{A,emp}$ and $\sigma_{AV,mle}^{2}$ *vs* $\sigma_{AV,emp}^{2}$). To allow for generalization to the population level, empirical and MLE-predictions for each participant were entered into one-sided paired t-tests (or one-sided Wilcoxon signed-rank tests if Kolmogorov–Smirnov tests indicated non-normal distributions) at the random effects group level. The tests were one-sided because (in addition to the fact that Alais and Burr (2004) also reported one-tailed tests) given our pilot data (Appendix A) and previously published reports (Battaglia et al., 2003), we expected that any difference would have been in the following direction: $w_{A,mle}>w_{A,emp}$ and/or $\sigma_{AV,emp}^{2}>\sigma_{AV,mle}^{2}$. Further assessments were made by computing one-sided Bayes factors using a Jeffreys prior on variance and a Cauchy prior on positive effect sizes for the alternative hypothesis (the prior is zero for negative effect sizes, interval c=[0,∞]; scaling factor r=√2/2) and a point prior on zero effect size for the null hypotheses (Rouder et al., 2009, Morey and Rouder, 2011; BayesFactor Package .9.12 in R 3.4.1; http://bayesfactorpcl.r-forge.r-project.org/). Bayes factor BF_01_ expresses evidence in favour of the null-hypothesis (no difference). BF_01_ > 3 indicates a good fit of the MLE model, whereas BF_01_ < $\frac{1}{3}$ indicates a significant difference between the empirical and MLE-predicted parameter values. Effect size index $d_{z}$ was computed as: (G*Power 3.1; www.gpower.hhu.de):

(7) $$d_{z}=\frac{\left|\mu_{x}-\mu_{y}\right|}{\sqrt{{\sigma_{x}}^{2}+{\sigma_{y}}^{2}-2\rho_{xy}\sigma_{x}\sigma_{y}}}$$

where $\mu_{x}$, $\mu_{y}$ and $\sigma_{x}$, $\sigma_{y}$ are the population means and standard deviations, and $\rho_{xy}$ denotes the correlation between the two measures.

### 2.10 Goodness of fit

The validity of the analysis method described above (Section 2.8, 2.9) relies on the assumption that the data for each condition can be accurately fitted by a cumulative Gaussian function. In order to validate this assumption we performed a goodness of fit test. This test compares i. the likelihood of participants' responses given the model that is constrained by the cumulative Gaussian function(s) to ii. the likelihood given a so-called ‘saturated’ model that models observers' responses with one parameter for each stimulus location in each condition. The likelihood ratio for the original data set is then compared with a null-distribution of likelihood ratios that is generated by parametrically bootstrapping data (5000x) from the model constrained by the cumulative Gaussian distribution (Kingdom and Prins, 2016, Wichmann and Hill, 2001b) and where additionally the expected probabilities of ‘right’ responses $\left({\bar{p}}_{i}\right)$ are drawn from beta distributions with mean $\psi \left(x_{i};a,\beta ,\lambda \right)$ and variance $\eta^{2}\psi \left(x_{i};a,\beta ,\lambda \right)\left(1-\psi \left(x_{i};a,\beta ,\lambda \right)\right)$ (Schütt et al., 2016). If fewer than 5% of the parametrically bootstrapped likelihood ratios were smaller than the likelihood ratio for the original data set (i.e., *p* < .05), then insufficient goodness of fit was inferred and the data set excluded (i.e., the participant was replaced). This exclusion criterion is required as parameters from psychometric functions that do not adequately fit observers' responses cannot be interpreted.

### 2.11 Summary of participant exclusion criteria

To ensure that our results and conclusions were based only on data sets from participants that maintain attention and provide reliable responses we have excluded participants prior to the final test session if i. their A localization performance was not adequate (accuracy < 90% for ±10° azimuth; Section 2.6.1.2), ii. the difference between unisensory auditory and visual variances was so large that the MLE predicted multisensory variance reduction was smaller than a third of the smallest unisensory variance (Section 2.6.1.4), iii. the lapse rate was greater than .06 or the goodness-of-fit was insufficient for either of the two unisensory psychometric functions obtained during the first session: A (Section 2.6.1.2) or V (Section 2.6.1.4). It is important to emphasize that participants were excluded from the study because of the above criteria prior to the main experiment which compared participants' audiovisual integration with the MLE predictions. In addition, we have excluded participants after the main experiment in the third session, if the lapse rate was greater than .06 or the goodness-of-fit was insufficient for the psychometric functions obtained during the main experiment (Sections 2.9, 2.10).

Excluded participants were replaced such that the final number of included participants was thirty-six (Section 2.3).

### 2.12 Summary of outcome-neutral conditions

The following criteria ensured that the data are of good quality, so that they enabled us to test the null-hypothesis that observers integrated audiovisual signals in line with MLE prediction: i. We included only participants with adequate auditory localization ability and performance (accuracy ≥ 90% for ±10° azimuth, Section 2.6.1.2) that were more likely to rely on their auditory sense during localization of AV stimuli. This will have excluded participants that may overweight the visual sense because auditory localization over an extended period of time is too demanding. ii. We have only included participants where we adjusted V reliability individually such that A and V perceptual reliability were approximately equated (Section 2.6.1.4). This criterion ensured that flooring/ceiling effects were avoided. It rendered our experimental design powerful for revealing a robust multisensory variance reduction if participants indeed integrated audiovisual signals according to MLE predictions (Eq (2), Section 2.1) and thus allowed us to dissociate whether or not human performance is in line with MLE predictions. iii. We have only included participants with lapse rates smaller than .06 (Wichmann & Hill, 2001b) and adequate goodness of fit (*p* > .05). This criterion ensured that data sets were included only from participants that consistently maintained attention and motivation throughout the entire experiment.

### 2.13 Post-hoc exploratory analyses

#### 2.13.1 Within-subject parameter comparisons

The fitted parameters for empirical weights and variances (see Section 2.9) are only estimates of observer's true weights and variances, because any psychometric function fit is inevitably affected by experimental noise (Kingdom & Prins, 2016). In order to visualize the amount of uncertainty that is associated with each estimate we made use of the parameter estimates that were fit during the goodness-of-fit parametric bootstrap procedure (N = 5000, see Section 2.10). 95% confidence intervals were computed as the distance between the 2.5 and 97.5 percentiles of the bootstrapped parameter distributions. Furthermore, using the bootstrapped distributions we tested whether observed differences between pairs of parameter estimates within the same participant were significant [e.g., (1) $\sigma_{V}\neq \sigma_{A}$, (2) $\sigma_{AV,emp}<min\left(\sigma_{A},\sigma_{V}\right)$, (3) $\sigma_{AV,emp}>\sigma_{AV,mle}$, and (4) $w_{A,emp}<w_{A,mle}$]: This was done by comparison of the empirically determined parameter difference with a null distribution of differences (i.e., expected differences due to noise when the two parameters are actually equal) which was constructed by subtracting the empirical difference from all bootstrapped parameter differences (thus ensuring that the null distribution is approximately centred at zero). Significance was inferred when the empirical difference exceeded 95% of the null-distribution (absolute values were used for two-sided tests).

#### 2.13.2 Control analysis for the effect of audiovisual spatial disparity and unisensory biases

If the forced fusion assumption does not hold in the cue conflict conditions, we may expect different variances for the audiovisual percept depending on the particular AV condition. To account for differences in AV variances, we performed a second psychometric function fit to all datasets, using the betabinomial model as described above. The only difference was that in this case five (instead of three) slope parameters were fitted, one for each condition (i.e., we fit $\sigma_{AV,emp}$ separately for each of the three audiovisual conditions). Moreover, using the parameter estimates of this second fit, we computed the auditory weights separately for the two incongruent conditions while taking unisensory biases into account:

(8) $$w_{A,ALVR}=\frac{PSE_{AV,ALVR}-\left(PSE_{V}-\frac{1}{2}X{}^{\circ}\right)}{\left(PSE_{A}+\frac{1}{2}X{}^{\circ}\right)-\left(PSE_{V}-\frac{1}{2}X{}^{\circ}\right)}\;\mathrm{and}\;w_{A,VLAR}=\frac{PSE_{AV,VLAR}-\left(PSE_{V}+\frac{1}{2}X{}^{\circ}\right)}{\left(PSE_{A}-\frac{1}{2}X{}^{\circ}\right)-\left(PSE_{V}+\frac{1}{2}X{}^{\circ}\right)}$$

where the abbreviation ALVR is used for the condition where the audiovisual spatial conflict is imposed as Auditory Left, Visual Right; i.e., ΔAV=+X°. Likewise VLAR is used for ΔAV=−X°.

## 3 Results

### 3.1 Participant exclusion and replacement

Five participants were excluded during/after the first session for the following reasons: (i) Two participants did not pass the unisensory auditory performance threshold (>90% at ±10°; Section 2.6.1.2). (ii) One participant was excluded because the difference between unisensory visual and auditory variances was too large even after they were supposedly matched using a staircase procedure [${\sigma_{AV,mle}}^{2}>\frac{2}{3}*\text{min}\left(\sigma_{A}^{2},\sigma_{V}^{2}\right)$; Section 2.6.1.4]. (iii) One participant was excluded because the unisensory visual lapse rate was too high (λ=0.11, Section 2.6.1.4). (iv) One participant was excluded in session 1 because the eye tracker failed to calibrate. Furthermore, two participants decided to withdraw from the study after successful completion of session 1. All seven participants were replaced such that thirty-six participants (26 women, 10 men; 21.8 mean age, ±2.6 years *SD*) completed all three sessions. All of these datasets were included for analyses (i.e., no dataset had to be excluded because the goodness of fit was inadequate or because the lapse rate was too high in the main experiment; Section 2.11).

### 3.2 Trial exclusion

Trials were excluded from analyses of the main experiment if the participant did not fixate within a 3° radius around the fixation cross or the participant blinked during either standard or probe stimulus presentation (Section 2.7). For six participants the collected eye tracker data were not reliable because of sudden jumps in gaze location and time gaps in which no data was collected. No trials were excluded for these six participants. For the other thirty participants we excluded on average 3% (maximally 12%) of the 2600 trials of interest of the main experiment (Section 2.6.2.2).

### 3.3 Main outcomes at the group level

**Fig. 2:**
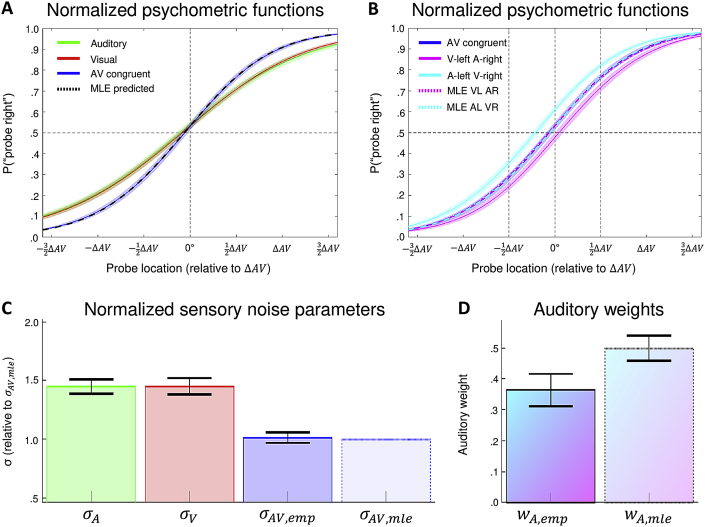
Psychometric functions, sensory noise parameters and weights – group level results. A-B. Psychometric functions were fit to responses for A, V and AV (congruent and incongruent) conditions of each participant. Panels A and B show the group-average of those fitted psychometric functions (group mean ±*SEM*) obtained by computing the mean P(“probe right”) across participants for every point on the x-axis (where the stimulus levels were expressed relative to the individual's ΔAV): Auditory (green), visual (red) and audiovisual congruent $AV_{\Delta =0{}^{\circ}}$ (blue), audiovisual conflict $AV_{\Delta =-X{}^{\circ}}$ (magenta; i.e., ‘visual left, auditory right’) and $AV_{\Delta =+X{}^{\circ}}$ (cyan; i.e., ‘auditory left, visual right’) as solid lines. Using individuals' MLE-predicted parameters (see Eqs. (1), (2)) we also constructed MLE-predicted psychometric functions and subsequently computed group-averages for $AV_{\Delta =0{}^{\circ}}$ (panel A, black), $AV_{\Delta =-X{}^{\circ}}$ (panel B, magenta) and $AV_{\Delta =+X{}^{\circ}}$ (panel B, cyan) in dashed lines. The empirical and MLE-predicted psychometric functions are nearly identical for the AV congruent condition. Furthermore, for the AV incongruent conditions the MLE-predicted psychometric functions are nearly identical to the empirical AV congruent psychometric function, because auditory and visual variances were approximately matched. By contrast, the empirical psychometric functions for the audiovisual incongruent conditions are shifted sideways, indicating more (less) “right” responses when the probe's visual signal was presented on the right (left) of the auditory signal. If participants completely ignored one of the two sensory modalities then their incongruent PSEs are expected to be near $\pm \frac{1}{2}\Delta \mathrm{AV}$ (vertical dashed black lines). C. Bar plots show the across participants' mean (±1.96 *SEM*) of the sensory noise parameter σ for the auditory (green), visual (red) and empirical (dark blue) and MLE-predicted (light blue, Eq. (2)) audiovisual conditions. The individual sensory noise parameters σ were normalized, i.e., divided by the participant-specific $\sigma_{\mathrm{AV},\mathrm{mle}}$ before averaging (hence $\sigma_{\mathrm{AV},\mathrm{mle}}=1$). D. Bar plots show the across participants' mean (±1.96 *SEM*) of empirical and MLE-predicted auditory weights (Eqs. (1), (6)).

We jointly fitted (using the betabinomial model, Eq. (5); Section 2.8) five psychometric functions individually to each participant's dataset of the main experiment: Unisensory auditory and visual, audiovisual spatially congruent and spatially incongruent (A-left V-right, ΔAV=+X°, and V-left A-right, ΔAV=−X°). Based on the unisensory variances we predicted (using MLE) the audiovisual variance and the PSEs of the incongruent audiovisual conditions (Eqs. (1), (2)). Fig. 2A,B summarizes the results at the group level. As expected under the MLE-model, the audiovisual slope (constrained to be equal for all three AV conditions) is steeper than either unisensory slopes and nearly identical to the MLE-predicted slope. Moreover, the PSEs (i.e., location at which $P\left("probe\;right"\right)=0.5$) for unisensory and AV congruent conditions are very similar (with a small bias for responding “probe right”, i.e., PSEs < 0°, for all three conditions). Because the auditory and visual variances were approximately matched (as intended, Section 2.6.1.3) the MLE-model predicts that the PSEs of the spatially incongruent conditions coincide with the AV congruent condition (Fig. 2B). By contrast, the empirical PSEs of the incongruent conditions deviate from the MLE-predicted PSEs. Both incongruent PSEs suggest that the visual stimulus component was assigned a stronger weight than expected based on MLE-predictions: e.g., when the visual and auditory probe were displaced by $+\frac{1}{2}\Delta AV$ and $-\frac{1}{2}\Delta AV$, respectively, this resulted in more “probe right” responses (thus a negative PSE shift; solid cyan line).

Fig. 2C,D shows the across participants' means (±*SEM*) for the MLE-predicted and empirical sensory noise parameters and auditory weights. In support of the MLE model we find no evidence that $\sigma_{AV,emp}>\sigma_{AV,mle}$ at the group level: *t*(35) = .33, *p* = .37, $BF_{01}$ = 4.24, $d_{z}$ = .06. However, the auditory weights are significantly smaller than predicted by the MLE model, $w_{A,emp}<w_{A,mle}$: *t*(35) = 6.25, *p* < .0001, $BF_{10}$ > 10000, $d_{z}$ = 1.04. (We reported results from one-sided t-tests because none of the Kolmogorov–Smirnov tests indicated that a non-parametric test was required.)

### 3.4 Post-hoc exploratory analyses

#### 3.4.1 Within-subject parameter comparisons

**Fig. 3:**
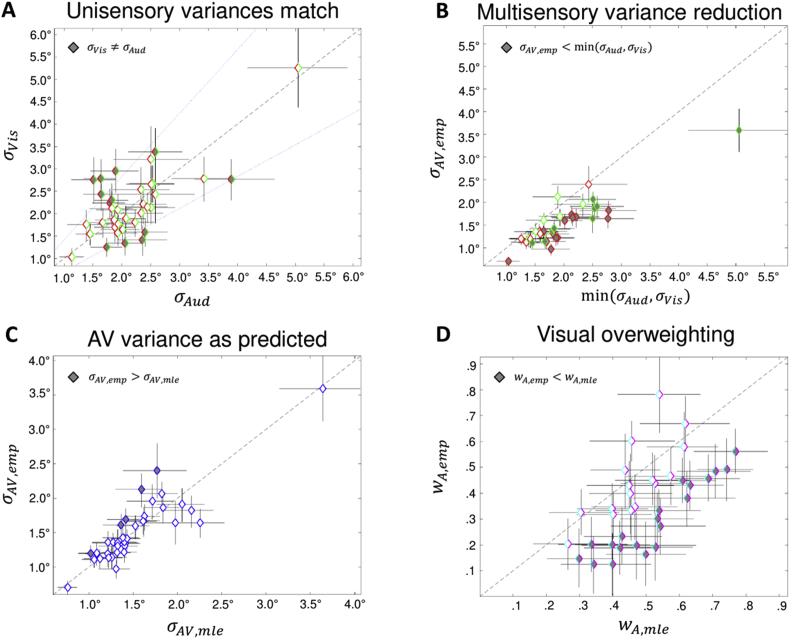
Scatter plots of individual sensory noise parameters and weights (with 95% bootstrapped confidence intervals for each parameter). Black dashed lines along the diagonal indicate equality of the two parameters. A. Unisensory visual (y-axis) versus auditory (x-axis) sensory noise parameters. Dark diamonds indicate twelve participants with a significant difference between auditory and visual sensory noise (two-sided test). B. Empirical audiovisual sensory noise parameters versus the minimum of the auditory or visual sensory noise parameters. The colour of the diamonds' outlines indicates the least variable unisensory modality for each participant (A = green, V = red). Dark diamonds indicate twenty-four participants with a significant multisensory variance reduction. C. Empirical versus MLE-predicted AV sensory noise parameters. Dark diamonds indicate five participants for whom the empirical variance is significantly greater than predicted by MLE. D. Empirical versus MLE-predicted auditory weights. Dark diamonds indicate twenty participants with significant visual overweighting.

The results reported so far clearly indicate that the MLE model is not an adequate description of observers' spatial classification responses. In the following we investigated individually for each participant whether his/her behavioural responses are consistent with the MLE predictions. Using parametric bootstrapping we obtained 95% confidence intervals for the estimates and performed statistical tests at the individuals level (Section 2.13.1). Fig. 3A shows that the visual reliability approximately matched the auditory reliability (see Section 2.6.1.3), though we acknowledge that they were significantly different in twelve participants. However, deviances from equality were only moderate, so that our experiment maintained its sensitivity for detecting differences with MLE-model predictions. Please note that the unisensory data for this statistical analysis were collected during the main experiment only (sessions 2–3).

Panel B shows that a significant multisensory variance reduction is observed for twenty-four participants, i.e., the majority of our participants [$\sigma_{AV}<min\left(\sigma_{A},\sigma_{V}\right)$]. (Panel C) While the one-sided bootstrap tests revealed an empirical audiovisual variance that is greater than predicted by the MLE model ($\sigma_{AV,emp}>\sigma_{AV,mle}$) for five participants, a smaller than predicted audiovisual variance was also found in a subset of participants. The fact that so few participants deviate from the MLE-predictions despite having optimized experimental conditions for finding such differences suggests MLE near-optimal multisensory integration. (D) However, we also observed that twenty participants significantly overweighted the visual signal during audiovisual integration ($w_{A,emp}<w_{A,mle}$), which clearly demonstrates that the group-level result for visual overweighting is not an accidental finding.

#### 3.4.2 Control analysis for the effect of audiovisual spatial disparity and unisensory biases

The analyses reported so far rest on two important assumptions: 1. The slope parameters of the three audiovisual conditions (independent of spatial in-congruence) are comparable and hence modelled jointly by one parameter when estimating the psychometric functions. 2. Any left-right bias detected in the unisensory auditory and/or visual condition is irrelevant when computing empirical auditory weights because left-right biases for audiovisual trials are best described by the PSE of the AV congruent condition. We therefore performed two additional control analyses that relaxed these assumptions:

1. We fitted psychometric functions using independent slope parameters for each of the three audiovisual conditions and investigated whether the audiovisual sensory noise parameters differed across the audiovisual congruent and the two conflict conditions. A one-way (AV congruent, AV conflict left, AV conflict right) repeated measures analysis on the fitted audiovisual noise parameters $\sigma_{AV,emp}$ revealed a significant effect of ‘audiovisual condition’ [*F*(1.63,57.19) = 5.97, *p* = .007, $\eta_{p}^{2}$ = .15, Greenhouse-Geisser corrected], with the order of the group means $\sigma_{AV,VLAR}<\sigma_{AV,congruent}<\sigma_{AV,ALVR}$. We also directly investigated whether the empirical audiovisual variances differed from their MLE-predictions separately for each AV condition. Indeed, one-sided paired t-tests revealed a significant difference for $\sigma_{AV,ALVR}>\sigma_{AV,mle}$: *t*(35) = 2.3, *p* = .04 (Bonferroni adjusted), $BF_{10}$ = 3.67, $d_{z}$ = .39. (No such difference was found for the other conditions: $BF_{01}$ ≈ 10 for both $\sigma_{AV,VLAR}>\sigma_{AV,mle}$ and $\sigma_{AV,congruent}>\sigma_{AV,mle}$). This suggests that the forced-fusion assumption has been violated in the spatially incongruent condition ALVR (ΔAV=+X°) leading to greater AV variances than predicted by MLE.

2. Using the fitted psychometric functions from point 1 above (with three audiovisual noise parameters), we computed the empirical auditory weights separately for the two incongruent conditions while accounting for the unisensory left-right bias (see Eq. (8), Section 2.13). This control analysis corroborated the visual overweighting for both incongruent conditions [$w_{A,VLAR}<w_{A,mle}$: *t*(35) = 3.30, *p* = .001, $BF_{10}$ = 31, $d_{z}$ = .55 and $w_{A,ALVR}<w_{A,mle}$: *t*(35) = 2.12, *p* = .02, $BF_{10}$ = 2.56, $d_{z}$ = .35].

#### 3.4.3 Results from the post-testing questionnaire

A critical assumption of cue conflict paradigms as used in the current study is that the cue conflict is non-noticeable for subjects, so that the forced fusion assumption holds. To assess whether this assumption holds in the current study we asked participants in a post-testing questionnaire whether they ever had the impression that the signals did not come from the same location (Section 2.6.2.3).

Only thirteen out of the thirty-six participants reported that they occasionally experienced the auditory and visual signals as coming from two separate locations on audiovisual trials (e.g., the auditory and visual component signals of the AV probe were perceived on opposite sides of the AV standard). These responses indicate an intermittent breakdown of the forced-fusion assumption in these thirteen participants on some trials. To investigate whether these thirteen participants showed a different pattern of results (relative to MLE-predictions) than the other twenty-three participants we performed two independent two-sample t-tests on (i) relative sensory noise differences $\sigma_{AV,emp}/\sigma_{AV,mle}$ and (ii) weights differences $w_{A,mle}-w_{A,emp}$ (as obtained using the primary analysis pipeline; Sections 2.8, 2.9). Both deviations from MLE-predictions were numerically larger in the group of thirteen participants who claimed to have experienced AV disparities, though neither of the tests reached significance: *t*(34) = 1.67, *p* = .052, Cohen's *d* = .58 for $\sigma_{AV}$, and *t*(34) = 1.49, *p* = .073, Cohen's *d* = .52 for $w_{A}$.

Furthermore, we investigated whether the visual overweighting was still present in the group that was not aware of the audiovisual conflict. Indeed, we still observed significant visual overweighting in the subgroup of twenty-three participants who did not report to have experienced any audiovisual disparity: *t*(22) = 3.70, *p* = .0006, $BF_{10}$ = 58.5, $d_{z}$ = .77. This finding suggests that visual overweighting in AV spatial localization is a general mechanism that does not critically depend on the awareness of the experimentally induced audiovisual disparity.

## 4 Discussion

This study investigated whether audiovisual localization is consistent with maximum likelihood estimation as previously suggested by Alais and Burr’s (2004). However, utilizing carefully designed methods that were peer-reviewed and registered before data collection, our study shows that naïve observers do not integrate signals as predicted by the MLE model. While we observed a significant variance reduction for audiovisual relative to unisensory conditions in agreement with MLE, participants weighted the visual signals significantly stronger than predicted by the MLE model. In the following we will first discuss differences between the current and the previous study by Alais and Burr (2004), we will then provide three explanations for the results observed in our study and finally elaborate on the most likely interpretation.

The design of this study has been optimized to allow detection of deviations from the MLE predictions with high statistical power. (i) We have recruited considerably more participants (N = 36) than were included in previous studies (i.e., Alais & Burr, 2004; N = 6; Battaglia et al., 2003, N = 10). (ii) We successfully matched the reliability of auditory and visual stimuli individually for each participant to sensitize our experimental design to the detection of deviations from the MLE model. (iii) We optimized audiovisual spatial disparity individually for each participant to balance the risk of violating the forced fusion assumption (small disparities are preferred) and high statistical power for potential weights differences (large disparities are preferred; Appendix B). (iv) We adjusted stimulus locations individually for each participant such that the parameters of the psychometric functions could be estimated reliably based on a high number of trials at relevant stimulus levels (i.e., spatial locations). (v) We ensured high quality data by excluding participants that showed signs of inadequate or inconsistent performance and by using eye tracking to control eye gaze fixation and remove missed trials due to blinks.

The beneficial effects of these optimized experimental conditions are best illustrated by the exploratory analyses at the level of the individuals (Fig. 3). We were able to demonstrate a significant audiovisual variance reduction (i.e., multisensory behavioural benefit) in two thirds of our participants, most likely because of relatively small confidence intervals for our parameter estimates (i.e., more reliable results). By contrast, Alais and Burr (2004) were able to demonstrate a significant variance reduction at the individual level in only one of their six participants. The observation of variance reduction at the individual level is critical to show that participants integrated audiovisual signals at the single trial level rather than probabilistically responding to either of the unisensory signals (i.e., they were not ‘cue-switching’; Ernst & Bülthoff, 2004). Yet, we also observed significant visual overweighting at the individuals-level in the majority of participants.

Moreover, post-hoc control analyses revealed visual overweighting independently in both audiovisual conflict conditions, so that this finding cannot be explained by unisensory biases. Finally, visual overweighting was significant at the group-level even when we constrained the analysis to the subset of participants who did not report the sensation of the audiovisual disparity (in a post-testing questionnaire).

Collectively these results suggest that observers do not integrate audiovisual signals into spatial representations consistent with the MLE model predictions. The dissociation between optimal variance reduction, yet suboptimal sensory weights may be surprising. However, we show in Appendix C that this dissociation is likely to emerge for small deviations from MLE optimality, because deviations from MLE prediction can be far more reliably estimated for sensory weights than for audiovisual variances. Thus, even though a reduction in audiovisual variance is critical to exclude probabilistic weighting as a potential strategy for observers, the confirmation of optimal sensory weights is equally important, because it will detect MLE sub-optimalities with a greater sensitivity.

In the following, we will present three explanatory frameworks that can accommodate our findings.

First, it is well-known that MLE-type integration of multisensory signals breaks down as a function of inter-sensory conflict, such as their spatial disparity (Gepshtein, Burge, Ernst, & Banks, 2005). More recent developments of Bayesian causal inference can accommodate this behavioural pattern by explicitly modelling the two causal structures that could have generated the two signals, i.e., common or independent sources (Körding et al., 2007, Rohe and Noppeney, 2015a, Rohe and Noppeney, 2015b, Rohe and Noppeney, 2016). A final perceptual (e.g., spatial) estimate is then obtained by combining the perceptual estimates obtained under the assumption of common and independent sources according to various decision functions. One particular decision function of the Bayesian causal inference model, the so-called ‘model-averaging’, combines the audiovisual common source estimate with the task-relevant (i.e., either auditory or visual) estimate weighted by the posterior probabilities of common or independent sources (Wozny et al., 2010, Rohe and Noppeney, 2015a). Crucially, observers may implicitly consider vision, that is usually the modality of choice for spatial localization, as the task-relevant modality, even though they were explicitly instructed to report the location of the audiovisual stimulus. Indeed, seven participants reported spontaneously in the post-testing questionnaire that they reported the visual location when they had the sensation that the A and V signals did not come from a common source. As a consequence of such a strategy, we would observe visual overweighting when comparing observers against the ‘inappropriate’ MLE model. However, because this question was not explicitly asked, the strategy of the other observers cannot be assessed.

Second, as previously suggested by Battaglia et al. (2003) observers may have assumed a prior over the visual signal's reliability based on their lifelong experience that vision is usually the more reliable sensory modality for spatial location. As a consequence their visual reliability estimates would be biased and observers would overweight visual signals when applying reliability-weighted audiovisual integration.

Both of the explanations discussed above, i.e., Bayesian causal inference and biased visual reliability estimates, predict not only visual overweighting, but also MLE violations for audiovisual variance. Yet, these violations may not have been observed in the current study, because the methodological approaches employed in cue conflict studies are far more sensitive for detecting violations in sensory weights than in sensory variances (see Appendix C).

Third, visual overweighting may emerge if the true visual reliability during visual-only trials is lower than the true visual reliability during audiovisual trials. For example, visual reliability might be higher in an audiovisual context because of low-level cross-modal boosting of stimulus salience (Aller, Giani, Conrad, Watanabe, & Noppeney, 2015) or because of sound-induced increased attention or vigilance (Talsma, Senkowski, Soto-Faraco, & Woldorff, 2010). Such an increase in the visual reliability during audiovisual presentation relative to the unisensory visual condition would increase the weight assigned to visual signals during multisensory integration. Because the MLE predictions assume that the visual variance during unisensory and audiovisual conditions are equal, observers' sensory weights and variances would deviate from the MLE predictions (that we based on the unisensory conditions). Specifically, visual weights would be greater and audiovisual variances smaller than predicted by MLE. Again, the variance deviations may not have been observed in our experiment because of lack of sensitivity.

In summary, our results demonstrate that observers integrate audiovisual signals approximately as predicted by MLE. However, the sensory weights deviated significantly from MLE predictions with a greater weight assigned to the visual signal. These findings converge with accumulating research revealing sensory overweighting not only for audiovisual spatial localization (Battaglia et al., 2003), but a range of different sensory modalities and tasks (Bentvelzen et al., 2009, Burr et al., 2009, Butler et al., 2010, Fetsch et al., 2009, Prsa et al., 2012, Rosas et al., 2005). We have proposed three possible accounts that can explain these modest deviations in sensory weights from MLE predictions. Below, we will elaborate on the most likely interpretation.

The fact that one third of the participants' did not always perceive audiovisual signals as coming from one source points to their causal uncertainty as key to understanding the sensory overweighting in our study. In everyday life observers are always confronted with uncertainty about the world's causal structure. Because of sensory noise even signals that come from a common source can be perceived as arising from different sources. Further, in our and previous perturbation studies a small intersensory conflict is required for the estimation of sensory weights, which may have further enhanced observers' uncertainty about the causal structure of the sensory signals. This produces a dilemma for experimentalists: Sensory weights are more sensitive to small deviations from MLE predictions than sensory variances. Yet, their estimation requires the introduction of intersensory conflicts that increase observer's causal uncertainty. By contrast, MLE predictions for sensory variances can be assessed based on congruent trials alone, but they are unreliably estimated making them an insensitive indicator for assessing whether observers' behaviour is consistent with MLE predictions. Thus, future studies may need to explore ways to increase observers' prior causal expectations, i.e., whether signals come from common or independent sources. Only if observers assume that sensory signals necessarily come from one source and hence adopt a causal prior of one, will the causal inference model reduce to the MLE model under forced fusion assumptions. For instance, future studies may include prior extensive training, increase the percentage of congruent stimuli and the persuasiveness of the stimuli (e.g., naturalistic voice and face pairings) to enhance observers' prior tendency to bind audiovisual signals according to forced fusion and hence MLE predictions.

In conclusion, naive observers do not integrate audiovisual spatial signals in a way that is consistent with the principles of maximum likelihood estimation. These results may be explained by observers' uncertainty about whether the sensory signals share a common origin, as accounted for by models of Bayesian causal inference.

## Open Practices

The study in this article earned Open Materials, Open Data and Preregistered badges for transparent practices. Materials and data for the study are available at https://osf.io/k3fj6/ (DOI 10.17605/OSF.IO/K3FJ6).

## Footnotes

2 The option for compensation by cash was added after stage 1 in-principle-acceptance (IPA) of the manuscript in order to recruit from a larger pool of potential participants.

3 The second ethics code was approved after IPA and is a replacement of the first ethics code.

4 In the stage-1 IPA version of this manuscript low contrast visual stimuli were said to be presented on a black background (1.9 cd/m^2^ on .12 cd/m^2^). The background was changed from black to grey after pilot testing revealed that the repeated abrupt changes in brightness caused participants discomfort.

5 Sound pressure level (SPL) was increased from 60 to 70 dB after post-IPA pilot testing to ensure that more participants could locate the auditory stimuli reliably.

6 The background story was changed after IPA to better match the type of sounds that were presented. Originally we told participants to think of a ball being thrown at the back of the screen.

7 This task was split into two parts after IPA and initial pilot testing. Performing the auditory reliability measurement using individualized probe locations (part 2) resulted in more precise estimates (see main text).

8 Exclusion of the estimate that was most distant from the mean was added to the protocol after post-IPA pilot tests had shown that it was fairly common for one of the six $\sigma_{blob}$ values to be an outlier.

9 The number of trials for visual reliability confirmation was reduced from 2x520 to 2x260 after IPA to reduce the overall duration of the first session and ensure that observers were able to maintain attention.

10 The type of eye tracker was changed after IPA. The Tobii EyeX gaming eye tracker (https://tobiigaming.com) that we had initially planned to use was upgraded to the Eyelink 1000 to provide more reliable eye movement recording data.

11 The in-principle-accepted version of this manuscript contained a different but equivalent equation for the likelihood as a product across all trials (instead of the current product across all locations). The change enables us to use identical parameters in Eqs (4), (5).

12 The betabinomial model (Eq. (5)) was adopted (instead of the binomial model, Eq. (4)) after in-principle acceptance of this registered report to improve model fits. The betabinomial model is currently the recommended procedure to account for modest overdispersion that we also observed in our data (η ≤ .13 for all participants). As a consequence, the betabinomial model substantially improved the goodness-of-fits, thereby ensuring reliable parameter estimates (Section 2.10). Control analyses that used the binomial model showed comparable results, i.e., deviations from MLE predictions were significant at the group-level for the empirical sensory weights but not significant for the AV variances in both analyses (see Sections 2.9, 3.3). This indicates that our results are robust to the specific model choice i.e., betabinomial versus binomial model.

13 The in-principle-accepted version of this manuscript contained a minor error in the weights equation (Eq. (6)). The signs of the PSEs of the incongruent conditions have been inverted to correct this error.
