# Supplementary material for: Integration of audiovisual spatial signals is not consistent with maximum likelihood estimation
Source: Cortex. 2019 Oct;119:74–88. doi: 10.1016/j.cortex.2019.03.026 (PMC6864592; doi:10.1016/j.cortex.2019.03.026)
Supplement: Multimedia component 1 [file mmc1.docx]

**[Supplementary Material – Appendices A, B, C]**

**Integration of audiovisual spatial signals is not consistent with maximum likelihood estimation**

David Meijer, Sebastijan Veselič, Carmelo Calafiore, Uta Noppeney

**Appendix A: Pilot study**

*Pilot study: Method*

Ten subjects participated in this pilot study. One participant was excluded because her $A$ localisation accuracy was below 90% at $10^{\circ}$ azimuth. Two other participants were excluded post-hoc, because they pressed random buttons in the latter half of the study’s experimental blocks. As a result, only seven participants (6 female, age range 18-20, all right handed) were included in the final analysis.

The experimental paradigm was comparable to the current research (main article), but differed in the following aspects: First, the visual stimulus that was used in the pilot study was a cloud of 20 dots (diameter: 0.43° visual angle) sampled pseudo-randomly from a bivariate Gaussian distribution (as in: Rohe & Noppeney, 2015a). Participants were told that the 20 dots were generated by one underlying source in the centre of the cloud. Second, the size of the visual cloud (i.e. spatial reliability) was not titrated per participant: horizontal standard deviation was$10^{\circ}$, vertical standard deviation was 3°. Third, the order of standard and probe stimulus was randomised over trials. Participants reported whether the first or second stimulus was more to the left. Fourth, the following 13 fixed locations were used: 0°, ±0.5°, ±1°, ±2.5°, ±5°, ±7.5°, ±10°. Fifth, the audiovisual disparity was fixed at ±5°.


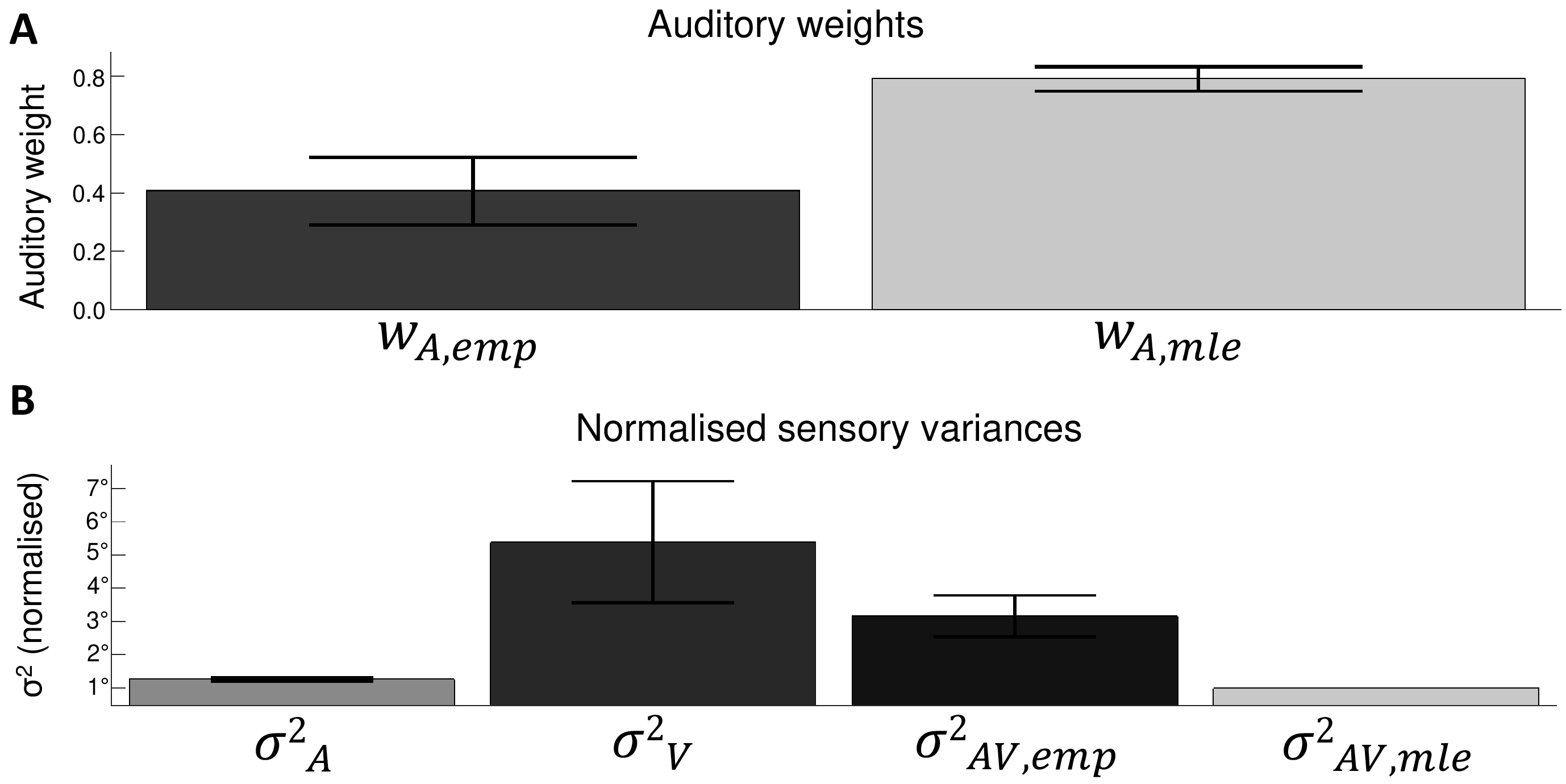
*Pilot study: Results*

**Fig A.1 - An overview of the most important pilot study results at the group level. The error bars depict the 95% confidence interval (**$\boldsymbol{\pm1.96*SEM}$**). A. Empirical and MLE-predicted auditory weights (**$\boldsymbol{w}_{\boldsymbol{A}}$**). B. Empirical and MLE-predicted sensory variances (**$\boldsymbol{\sigma}^{\boldsymbol{2}}$**). Before averaging across participants, the sensory variances of the individual participants were normalised with respect to**${\boldsymbol{\sigma}^{\boldsymbol{2}}}_{\boldsymbol{AV, mle}}$ **(for illustration purposes only).**

The most important results of the pilot study are shown in Fig A.1. Although the $A$ reliability was greater than the $V$ reliability in all participants (non-normalised group-level means: $\sigma_{V}$ = 3.5°, $\sigma_{A}$ = 1.77°), participants did not weight the more reliable $A$ stimuli according to MLE predictions (Eq. 1, Section 2.1) in the $AV$ context ($w_{A,emp}$ < $w_{A,mle}$ for all participants). The group-level Wilcoxon signed rank test demonstrated a significant difference between the empirical and MLE-predicted auditory weights (*p* = .0078, $d_{z}$ = 2.99). While the variance of the $AV$ conditions decreased relative to the unisensory $V$ condition, we observed no multisensory benefit relative to the more reliable unisensory $A$ condition ($\sigma_{A}$ < $\sigma_{AV,emp}$ for all participants). In line with this, the empirical variance for the $AV$ conditions was significantly greater than predicted by the MLE model (Eq. 2, Section 2.1; *p* < .05 for all participants). The group-level Wilcoxon signed rank test demonstrated a significant difference between the empirical and MLE-predicted $AV$ variances (*p* = .0078, $d_{z}$ = 3.17).

Importantly, the goodness of fits were sufficient for all seven participants (*p* > .05) and no lapse rate was greater than 0.06. We conclude that although the empirical auditory weights are quite high for an audiovisual localisation task (mean $w_{A, emp}$ = 0.41), we did not observe the MLE-predicted and previously reported ‘reverse ventriloquist effect’, i.e. a shift of the perceived $AV$ location toward the auditory signal on cue conflict trials (Alais & Burr, 2004). Instead, during audiovisual integration, all participants overweighed their less reliable visual modality, similar to the results of Battaglia et al., (2003).

**Appendix B: Selection of audiovisual disparity individually for each participant based on power analysis**

Observers’ sensory weights are estimated empirically by introducing a small conflict between the sensory cues – a procedure coined ‘perturbation analysis’ (Young, Landy & Maloney, 1993). This raises the question of how to select the conflict size (e.g. audiovisual disparity for spatial localization). On the one hand a greater conflict size is preferable, because it renders the perturbation analysis more sensitive for detecting deviations of observers’ empirical weights from MLE predictions. On the other hand, greater conflict sizes may prevent participants from integrating sensory signals into one unified percept according to forced fusion assumptions. Instead, observers are then likely to compute a perceptual estimate that takes into account the uncertainty about the world’s causal structure as accommodated by more complex models of causal inference (see Körding, Beierholm, Ma et al., 2007; Shams & Beierholm, 2010; Rohe & Noppeney, 2015a, 2015b, 2016). Moreover, previous research has shown that observers’ sensitivity to intersensory conflicts depends on their perceptual reliability (Rohe & Noppeney, 2015a). Collectively, these considerations suggest that i. we need to determine the minimal conflict size (i.e. here: spatial disparity) that enables detections of deviations of the empirical sensory weights from MLE predictions with high statistical power (e.g. 1-β = 0.95) and that ii. we need to adjust this minimal conflict size individually for each participant based on their unisensory perceptual reliability as indexed by their auditory JND.

To determine the minimal conflict size (ΔAV in standardized JND units) that still enables detection of deviations of observers’ empirical weights from MLE predictions with high statistical power, we performed the following simulations:

1. For each of the 36 participants, we initially sampled an auditory JND from a uniform distribution between 1.2 and 3.8° visual angle, i.e. a range of auditory JNDs that we usually observe for our audiovisual experimental set up and stimuli across participants. Exactly as in the current study, we set the visual JND equal to the auditory JND. The PSE of the A and V conditions were set to zero, i.e. we assumed no perceptual biases. The audiovisual JND and PSE were set to the MLE predicted values computed from the unisensory JNDs and PSEs according to Eq. 1 and Eq. 2 (Section 2.1); i.e. the PSE of the audiovisual condition was also set to zero. Hence, the ‘true’ empirical weights (to be computed from the $AV$ PSEs) and the MLE-predicted weights (to be computed from the unisensory $A$ and $V$ JNDs) are by construction all equal to 0.5; reflecting equal perceptual reliabilities of the $A$ and $V$ signals. Likewise, by construction the difference between the ‘true’ empirical and MLE-predicted auditory weights is zero. To assess the variability (or uncertainty) of this difference between empirical and MLE-predicted auditory weight estimates, and how this variability will depend on the conflict size (i.e. spatial disparity ΔAV) we generate distributions of empirical and MLE-predicted weights as follows:

2. For each participant, we parametrically bootstrap (Palamedes toolbox 1.8.2, Kingdom & Prins, 2016) 1000 $A$ and $V$ data sets (with lapse rate parameter (λ) set to 0.02), stimulus locations set according to the subject-specific auditory JND (see Section 2.6.1.2) and 40 trials per location), and we subsequently fit psychometric functions to each simulated data set. From the fitted unisensory JNDs we compute the MLE-predicted auditory weights according to Eq. 1. This will generate a distribution of one thousand MLE-predicted auditory weights centred on 0.5, for each participant.

3. Likewise, we sample 2 x 1000 $AV$ data sets and fit an $AV$ psychometric function to each simulated data set. In order to evaluate the effect of spatial disparity on the precision of the estimated empirical auditory weights (and as a consequence also on the differences between empirical and MLE-predicted weights) we now arbitrarily assume that half of the $AV$ data sets were generated by a positive conflict ${AV}_{\Delta=+X^{\circ}}$ and the other half by a negative conflict ${AV}_{\Delta=-X^{\circ}}$. Empirical auditory weights are then computed based on Eq. 6 (Section 2.9) for a range of spatial disparity sizes in participant’s auditory JND units (i.e. $\Delta AV=factor*JND$; with ‘factor’ logarithmically sampled in 50 steps from between 0.1 and 2). For each participant, this will generate a distribution of one thousand empirical auditory weights per spatial disparity $\Delta AV$. By construction, the distributions of empirical weights are all centred on 0.5. Yet importantly, they vary in their spread: the larger the spatial disparity, the smaller the spread of the distribution of empirical weights (as follows directly from Eq. 6).

4. Finally, we shift the distributions of empirical auditory weights by subtracting a ‘true’ (i.e. to be detected) variable value (range 0 – 0.25); thereby creating empirical weights distributions that are no longer centred at 0.5 (i.e. we ‘simulate’ visual overweighting). Critically, while the difference in means between i. the distributions of the MLE-predicted weights and ii. the shifted distribution of empirical auditory weights is equal to this specific value irrespective of spatial disparity, the variance of the empirical weight distribution and hence the overlap of the two distributions depends on the spatial disparity ($\Delta AV$).

5. To enable a power analysis at the random effects group-level, we enter one bootstrapped pair of MLE-predicted and empirical weights (with specific subtracted ‘true’ difference and spatial disparity level $\Delta AV$) for each participant into one-sided paired t-tests (or one-sided Wilcoxon signed-rank tests if Kolmogorov-Smirnov tests indicated non-normal distributions). These one-sided group-level paired t-tests for each level of spatial disparity and imposed ‘true’ difference between empirical and MLE-predicted weights are then repeated for each of the 1000 bootstraps. As a result of this procedure, we can compute the fraction of bootstraps (i.e. experiments) where the paired t-test successfully declares the empirical auditory weights for a particular ‘true’ difference as significantly smaller than the MLE-predicted auditory weights (*p* < .05). In other words, we compute the power of the statistical test separately for each combination of ‘true’ difference and spatial disparity.


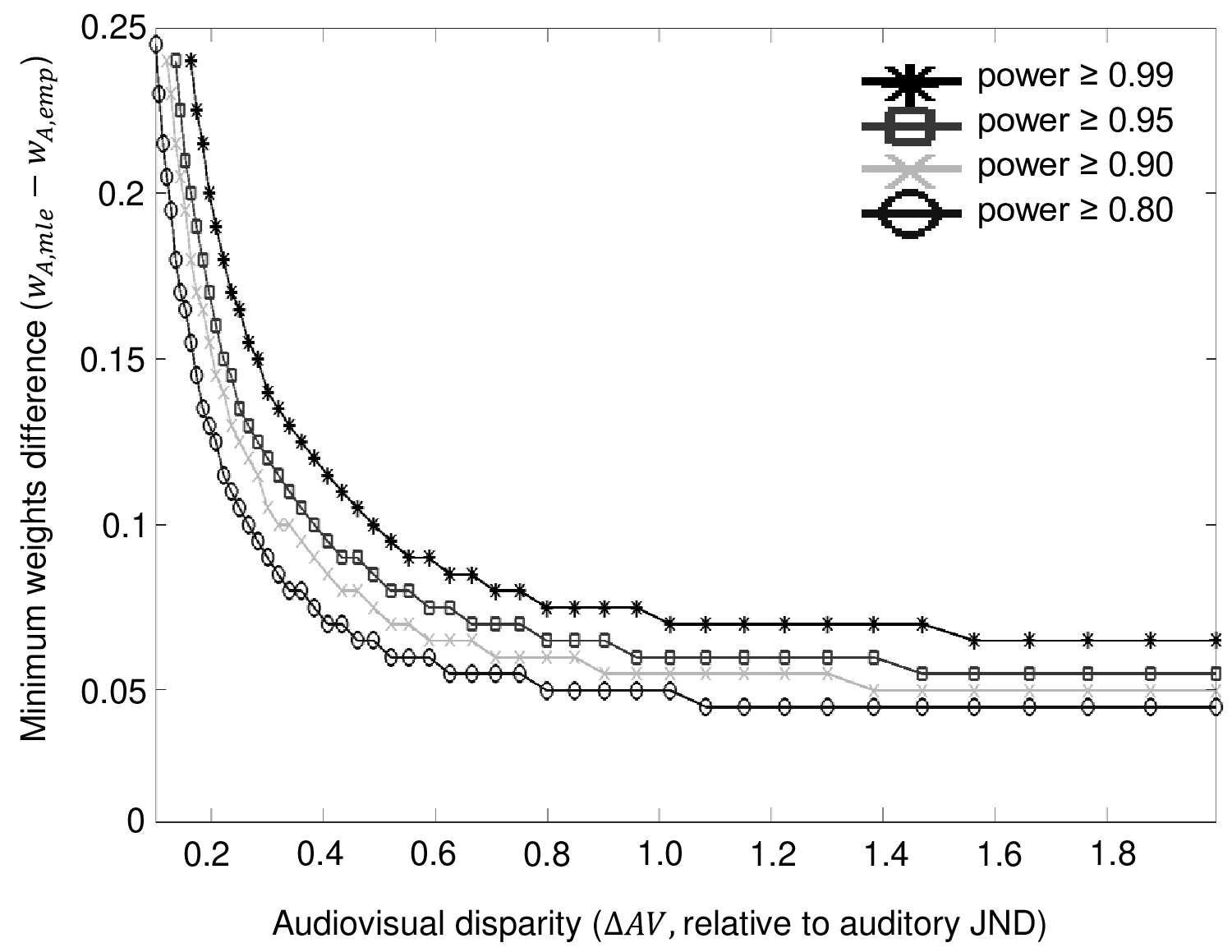


**Fig B.1 – Results of power analysis simulations for selecting spatial disparity: Minimum deviations of empirical weights from MLE-predicted weights (**$\boldsymbol{w}_{\boldsymbol{A,mle}}\boldsymbol{\approx0.5}$**) that are detected with a power of ≥0.8 (circles), ≥0.9 (crosses), ≥0.95 (squares), ≥0.99 (asterisks) as a function of AV spatial disparity (in standardized subject-specific auditory JND units).**

Fig. B.1 shows the minimum ‘true’ difference between empirical and MLE-predicted auditory weights that can be detected with a power of ≥0.8, ≥0.9, ≥0.95, ≥0.99 as a function of spatial disparity (in JND-standardized units). The results from this power analysis suggest that for our study with 36 subjects, specific parameter choices and for a spatial disparity equal to an individual’s auditory JND (i.e. in Fig B.1, ∆AV = 1 in standardized JND units) a difference in empirical and MLE-predicted auditory weights of 0.06 would be detected with a power of 0.95. Critically, while the minimal difference between empirical and MLE-predicted weights that can be detected with 0.95 power rapidly increases for AV disparities smaller than one subject-specific auditory JND, AV disparities greater than one auditory JND have negligible impact on the power of perturbation analyses for our experimental choices.

**Appendix C: Simulating visual overweighting with apparent MLE-optimal audiovisual variance**

This study (main text) suggests that participants assigned a significantly greater weight to the visual signals than predicted by MLE ($w_{A,emp}<w_{A,mle}$). Nevertheless, the audiovisual variance was not significantly different from the MLE predicted variance (${\sigma^{2}}_{AV, emp}\approx{\sigma^{2}}_{AV, mle}$), i.e. observers showed the predicted multisensory variance reduction. In the following, we will use Monte Carlo simulations to show that this seemingly contradictory dissociation between MLE-optimal variances and MLE-suboptimal sensory weights can result from differences in the precision with which current methodological approaches based on psychometric function fitting estimate an observer’s variances and weights (i.e. PSE).

We simulated MLE-suboptimal audiovisual integration behaviour for thirty-six observers using the experimental parameters of the current study. We then fitted psychometric functions to these simulated observers’ responses and show that, for moderate levels of visual overweighting, the group-level statistical outcomes are similar to the results reported in the main manuscript (i.e. MLE optimal variance despite sensory weights that deviate significantly from MLE predictions). The methodological steps are as follows:

1. For each of the thirty-six simulated observers, the individualized probe locations $S_{pr}$ and audiovisual disparity $\Delta AV$were set to the locations and disparity used for one of the thirty-six participants of the current study.

2. For each observer, we simulated forty sensory measurements per probe location for each of the five stimulus conditions ($A$, $V$, ${AV}_{\Delta=0^{\circ}}$, ${AV}_{\Delta=+X^{\circ}}$, ${AV}_{\Delta=-X^{\circ}}$) as follows:

For each 2IFC trial, we independently drew $x_{A}$ and/or $x_{V}$ from a Gaussian distribution for the standard as well as the probe stimulus. The Gaussian distribution was centered on the true auditory and/or visual locations (n.b. $S_{A}\neq S_{V}$ in the incongruent AV conditions). The variance was set to the participant-specific unisensory noise parameters $\sigma_{A}$, $\sigma_{V}$ (that were estimated in the main paper by fitting psychometric functions to participants’ responses):

$x_{A} \sim N(S_{A},{\sigma_{A}}^{2})$ and/or $x_{V} \sim N(S_{V},{\sigma_{V}}^{2})$

3. For unisensory conditions, we assumed that observers responded ‘right’ if

$x_{pr}-x_{st}>0^{\circ}$ with $x_{pr},x_{st}$ both being $x_{V}$ (resp. $x_{A}$)

For audiovisual conditions, we assumed that observers responded ‘right’ if

$\left( w_{V,sub}x_{V,pr}+w_{A,sub}x_{A,pr} \right)-\left( w_{V,sub}x_{V,st}+w_{A,sub}x_{A,st} \right)>0$

To simulate a suboptimal observer that overweights vision, we computed the suboptimal weights as:

$w_{V,sub}=\frac{{\sigma_{A}}^{2}}{(1-\pi){\sigma_{V}}^{2}+{\sigma_{A}}^{2}}$ and $w_{A,sub}=1-w_{V,sub}$

With $\pi$ (between 0 and 1) as a factor to simulate visual overweighting (c.f. Battaglia et al., 2003).

4. For each simulated observer, we jointly fitted five ($A$, $V$, ${AV}_{\Delta=0^{\circ}}$, ${AV}_{\Delta=+X^{\circ}}$, ${AV}_{\Delta=-X^{\circ}}$) psychometric functions to the binary ‘right’ responses from step 3, exactly as described in the methods section of the main paper (see Section 2.8). The slope parameters (n.b. $\frac{1}{\beta}=\sqrt{2}\sigma_{sim}$) that we obtained from these fitted auditory and visual psychometric functions were then used to compute the MLE-predicted weights and audiovisual variance (i.e. Equations 1-2 from the main paper):

$w_{V,sim,mle}=\frac{{\sigma_{A,sim}}^{2}}{{\sigma_{V,sim}}^{2}+{\sigma_{A,sim}}^{2}}$ and $w_{A,sim,mle}=1-w_{V,sim,mle}$

$\sigma_{AV,sim,mle}=\sqrt{\frac{{\sigma_{A,sim}}^{2}{\sigma_{V,sim}}^{2}}{{\sigma_{V,sim}}^{2}+{\sigma_{A,sim}}^{2}}}$

The empirical audiovisual variance ($\sigma_{AV,sim,emp}$) was obtained from the fitted audiovisual slope parameter. The empirical auditory weight was computed according to Equation 6 based on the PSEs that were obtained from fitting a psychometric function to the simulated responses for the two spatially incongruent audiovisual conditions:

$w_{A,sim,emp}=\frac{{PSE}_{\Delta AV= +X^{\circ},sim}-{PSE}_{\Delta AV= -X^{\circ},sim}}{2*|\Delta AV|}+\frac{1}{2}$

5. Using one-sided paired t-tests at the random effects group level (i.e. across all thirty-six simulated observers, significance threshold *p* < .05) we investigated whether the simulated empirical weights (resp. variances) deviated significantly from their MLE-predictions: $w_{A,sim,mle}>w_{A,sim,emp}$ and/or$\sigma_{AV,sim,emp}>\sigma_{AV,sim,mle}$ (c.f. Section 2.9).

6. To simulate multiple experiments, we repeated steps 2-5 one thousand times for each of 32 different values of visual overweighting factor $\pi$ (regular intervals between $\pi$ = 0 and $\pi$ = 0.31). For each setting of $\pi$ we computed the proportion of the 1000 experiments where we observed significant deviations from the MLE-predictions, separately for weights and AV variances (i.e. step 5).

Figure C.1 (panel A) shows the results of these simulations. Significant deviations of the empirical sensory weights from MLE-predictions can be detected with a statistical power of >0.9 even for small visual overweighting (i.e. $\pi\geq0.08$). By contrast, significant deviations of audiovisual variance from MLE-predictions can be detected with a statistical power of >0.9 only for $\pi\geq0.26$. Put differently, for a particular level of visual overweighting, deviations of sensory weights from MLE predictions can be detected with a greater statistical power than deviations of audiovisual variances from their MLE predictions.


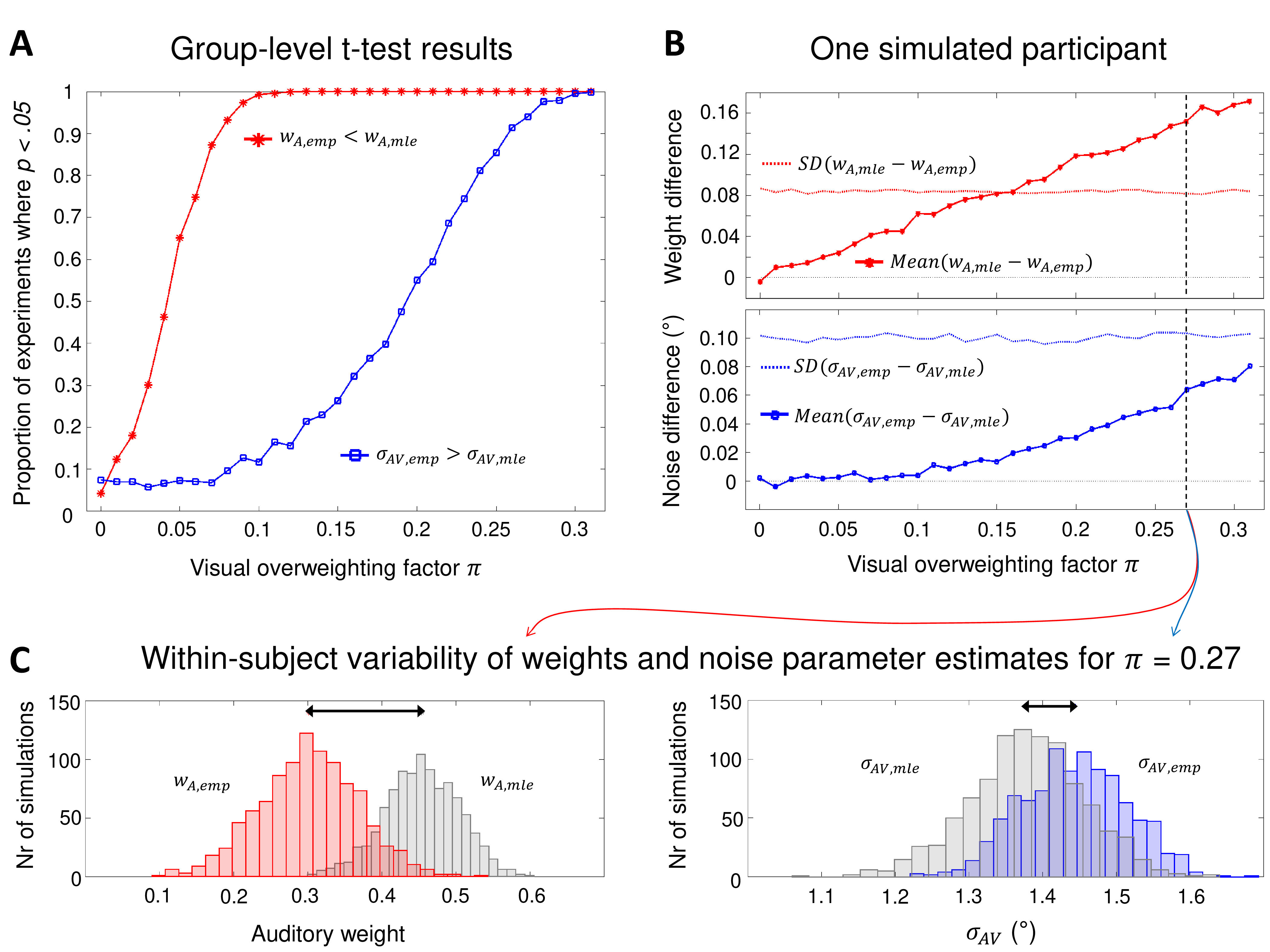
Moreover, these differences in sensitivity for detecting deviations from MLE predictions for sensory weights and audiovisual variances at the group level can be explained by differences in the precision with which PSEs (i.e. sensory weights) and slope parameters (i.e. audiovisual variance) of the psychometric functions are estimated. For a simulated observer (with $\pi=0.27$, $\sigma_{A}=2.1$, $\sigma_{V}=1.9)$ we observed a greater variability and hence greater overlap of the empirical and MLE-predicted distributions of audiovisual sensory noise ($\sigma_{AV,sim}$) as compared to sensory weights (across 1000 simulated experiments; Figure C.1, panel C). This is also illustrated when comparing the mean and standard deviation (across experiments) of the difference between empirical and MLE-predicted sensory weights (top panel) and audiovisual sensory noise (bottom panel) for different levels of visual overweighting (Figure C.1, panel B).

**Fig C.1 – Simulation results for deviations of empirical sensory weights and sensory noise parameters from MLE predictions. A. The proportion of 1000 simulated experiments with 36 suboptimal observers that revealed significant deviations of the empirical auditory weights (i.e.** $\boldsymbol{w}_{\boldsymbol{A,sim,emp}}$**, red) and sensory noise parameters (i.e.** $\boldsymbol{\sigma}_{\boldsymbol{AV,sim,emp}}$**, blue) from their MLE-predictions at the group-level (*p* < .05) is shown as a function of visual overweighting factor** $\boldsymbol{\pi}$**. B. For one simulated suboptimal observer, the differences (mean and SD across 1000 simulations) between the empirical and MLE-predicted AV noise parameters (top panel) and auditory weights (bottom panel) are shown as a function of visual overweighting factor** $\boldsymbol{\pi}$**. C. For the same representative suboptimal observer (with** $\boldsymbol{\pi=0.27}$**, indicated by black dashed line in panel B), the histograms of the 1000 simulated empirical (colored) and the corresponding MLE-predicted (grey) auditory weights (left panel) and audiovisual noise parameters (right panel) are shown. The difference between the across-simulations’ means of empirical and MLE-predicted parameters is illustrated by the arrow on top of the distributions.**

**References**

Alais, D., & Burr, D. (2004). The Ventriloquist Effect Results from Near-Optimal Bimodal Integration. *Current Biology, 14*(3), 257–262. https://doi.org/10.1016/j.cub.2004.01.029

Battaglia, P. W., Jacobs, R. A., & Aslin, R. N. (2003). Bayesian integration of visual and auditory signals for spatial localization. *Journal of the Optical Society of America A, Optics, Image Science and Vision, 20*(7), 1391-1397.

Kingdom, F. A. A., & Prins, N. (2016). *Psychophysics* (Second Edition). San Diego: Academic Press. http://www.sciencedirect.com/science/book/9780124071568

Körding, K. P., Beierholm, U., Ma, W. J., Quartz, S., Tenenbaum, J. B., & Shams, L. (2007). Causal Inference in Multisensory Perception. *PLoS ONE, 2*(9), e943. https://doi.org/10.1371/journal.pone.0000943

Rohe, T., & Noppeney, U. (2015a). Sensory reliability shapes perceptual inference via two mechanisms. *Journal of Vision, 15*(5), 22. https://doi.org/10.1167/15.5.22

Rohe, T., & Noppeney, U. (2015b). Cortical Hierarchies Perform Bayesian Causal Inference in Multisensory Perception. *PLOS Biology, 13*(2), e1002073. https://doi.org/10.1371/journal.pbio.1002073

Rohe, T., & Noppeney, U. (2016). Distinct Computational Principles Govern Multisensory Integration in Primary Sensory and Association Cortices. *Current Biology, 26*(4), 509–514. https://doi.org/10.1016/j.cub.2015.12.056

Shams, L., & Beierholm, U. R. (2010). Causal inference in perception. *Trends in Cognitive Sciences, 14*(9), 425–432. https://doi.org/10.1016/j.tics.2010.07.001

Young, M. J., Landy, M. S., & Maloney, L. T. (1993). A perturbation analysis of depth perception from combinations of texture and motion cues. *Vision Research, 33*(18), 2685–2696.
